# Prevalence of Erectile Dysfunction in Patients With Abdominal Aortic Aneurysm: An Exploratory Study

**DOI:** 10.3389/fcvm.2022.847519

**Published:** 2022-02-28

**Authors:** Gianmarco de Donato, Edoardo Pasqui, Bruno Gargiulo, Giulia Casilli, Giulia Ferrante, Giuseppe Galzerano, Alessandro Cappelli, Giancarlo Palasciano

**Affiliations:** Vascular Surgery Unit, Department of Medicine, Surgery and Neuroscience, University of Siena, Siena, Italy

**Keywords:** erectile dysfunction, vasculogenic impotence, abdominal aortic aneurysms, endovascular aneurysm repair, peripheral artery disease

## Abstract

**Introduction:**

Erectile dysfunction (ED) is defined as the recurrent inability to achieve and maintain a satisfactory erection for sexual intercourse. Many studies have highlighted that ED shares common cardiovascular risk factors with cardiovascular disease. No data are reported about the prevalence of ED in patients with the abdominal aortic aneurysm (AAA). The aim of our study was to investigate the preoperative information given about sexual functions of patients undergoing endovascular aneurysm repair (EVAR) and to compare it with the presence and severity of steno-occlusive atherosclerotic lesions of the pelvic arterial tree at pre-operative Computed Tomography Angiography (CTA).

**Methods:**

We prospectively enrolled all men patients who underwent elective EVAR from September to November 2021. Preoperative ED was evaluated using the International Index of Erectile Function (IIEF-5) questionnaire. Preoperative imaging was routinely performed with CTA scan of the abdominal aorta and iliac-pelvic district. An innovative score of pelvic arterial disease associated to AAA was defined, dividing the iliac district in 4 zones attributing a grading of severity for each zone bilaterally (score ranges 0–24). Linear regression analysis was used to correlate IIEF-5 score to anatomical score of pelvic arterial steno-occlusive disease.

**Results:**

A total of 25 patients were enrolled. Median age was 74 ± 5.3 years. IIEF-5 average score was 14.8 ± 7.1. Eight cases (32%) had severe ED; one case (4%) had moderate, five patients (20%) had mild to moderate ED; five patients (20%) had mild ED, and 6 (24%) patients had no ED. CTA evaluation revealed an average anatomical score of 7.9 ± 4.5. Pelvic disease was considered moderate-severe in 20 cases (80%) and not significant in 20% (five cases). Linear regression analysis confirmed the hypothesis that a more diseased pelvic arterial tree was correlated to a more severe ED (*Y* = −1.531^*^ × + 26.35 [slope *CI*: −1.946 to −1.117, *p* < 0.0001]).

**Conclusion:**

Although typically unreported, the prevalence of ED associated to AAA was found to be high. A vasculogenic origin of ED in patients with AAA is plausible and may be easily confirmed by the evaluation of pelvic arterial distribution at angio-CT performed for EVAR planning. Our proposed “MAPPING AND SCORING SHEET” may help to identify the vasculogenic origin of ED in AAA patients.

## Introduction

Erectile dysfunction (ED) is defined as the recurrent inability to achieve and maintain a satisfactory erection for sexual intercourse (1). Although the delicate and sensitive dimension of this condition do not allow to clearly define its real prevalence, it is estimated that more than 150 millions of men suffered from ED (2), 52% of men aged 40–70 have some degree of ED, and moderate to severe cases increase sharply with age (3). The etiologies are several and could be complex. One of the most notable is the vasculogenic origin, determined by the presence of arterial inflow atherosclerotic lesions, or cavernosal smooth muscle dysfunction or veno-occlusive insufficiency. Many studies have highlighted that ED shares common cardiovascular risk factors with coronary artery disease (CAD), peripheral artery disease (PAD), and carotid artery disease especially in case of arterial inflow impairment (4).

Little is known about the prevalence of ER in patients with abdominal aortic aneurysms (AAA) before repair. Open repair can injury the autonomic nerve and affect sexual functions negatively, resulting in a negative impact on the quality of life, while the incidence of sexual dysfunction is lower overall in patients operated upon with endovascular aortic repair (EVAR) (5). Although often unreported, the ED can be presented already before EVAR because of the presence of arterial inflow atherosclerotic lesions of pelvic area.

The aim of the study was to investigate the prevalence of ED through the preoperative information given about sexual functions of patients undergoing EVAR, and to compare it with the presence of steno-occlusive atherosclerotic lesions of the pelvic arterial tree at pre-operative CT.

## Materials and Methods

### Patients' Population

We prospectively enrolled all men patients admitted to our Department of Vascular Surgery to undergo elective EVAR from September 2021 to November 2021.

Indication to EVAR was AAA diameter of >5.5 cm in man and >5 cm in woman, or if the aneurysm was rapidly increasing (>0.5 cm in 6 months or >1cm in 12 months). All patients treated in an urgent or emergent setting and all patients treated for other conditions than AAA (such as dissection and iliac aneurysm) were excluded. Patients evaluated for EVAR with a CT without contrast were also excluded from the enrollment.

Patients' demographics were prospectively compiled in a dedicated database with preset variables. Hypertension, CAD, diabetes mellitus (DM), chronic obstructive pulmonary disease (COPD), renal disease (chronic renal insufficiency defined by serum creatinine level >1.2 mg/dl), smoking history (any current or past regular use of tobacco), congestive heart failure (CHF), history of cerebrovascular events (stroke and/or transient ischemic attacks), history of cancer (any current or past incidence if malignancy), atrial fibrillation (AF), and dyslipidemia were taken in account as comorbidities.

Drugs assumption was also evaluated, with a major attention relatively to their eventual impact on sexual function.

The study followed the Declaration of Helsinki on medical protocol. Patient informed consent for data collection was obtained, and the local institutional review board was informed of the descriptive, non-experimental nature of the study.

### Erectile Dysfunction Evaluation

At the time of the admission, the International Index of Erectile Function-5 (IIEF-5) (6, 7) was submitted to all patients. The questionnaire is made by five questions; each IIEF-5 item is scored on a five-point ordinal scale where lower values represent poorer sexual function. Thus, a response of 0 for a question was considered the least functional, whereas a response of five was considered the most functional. The possible scores for the IIEF-5 range from 5–25 (one question has scores of 1–5), and a score above 22 was considered as normal erectile function, and at or below this cutoff was considered as ED. According to this scale, ED is classified into four categories based on IIEF-5 scores: severe (5–7), moderate (8–11), mild-to-moderate (12–16), mild (17–21), and no ED (22–25). Table 1 reports the IIEF-5 questions and answers. Trained physicians interviewed each of the men in an individual room with a total guarantee of confidentiality.

**Table 1:** International Index of Erectile Function-5 (IIEF-5) questionnaire.

**Over the past 6 months:**
Q1: How do you rate your confidence that you could get and keep an erection?
1. Very Low 2. Low 3. Moderate 4. High 5. Very High
Q2: When you had erections with sexual stimulation, how often were your erections hard enough for penetration?
1. Almost never/never 2. A few times (much less than half the time) 3. Sometimes (about half the time) 4. Most times (much more than half the time) 5. Almost always/always
Q3: During sexual intercourse, how often were you able to maintain your erection after you had penetrated (entered) your partner?
1. Almost never/never 2. A few times (much less than half the time) 3. Sometimes (about half the time) 4. Most times (much more than half the time) 5. Almost always/always
Q4: During sexual intercourse, how difficult was it to maintain your erection to completion of intercourse?
1. Extremely difficult 2. Very difficult 3. Difficult 4. Slightly difficult 5. Not difficult
Q5: When you attempted sexual intercourse, how often was it satisfactory for you?
1. Almost never/never 2. A few times (much less than half the time) 3. Sometimes (about half the time) 4. Most times (much more than half the time) 5. Almost always/always

Moreover, we investigated whether the patient had ever discussed about sexual issue with their healthcare provider or with any other medical specialists.

### Anatomical Evaluation

Preoperative imaging was routinely performed with an angio-CT (GE Healthcare, discovery 750 hd) scan of the abdominal aorta and iliac district of 1.25 mm slice thickness.

The CT exam was performed using a standardized protocol (recin int 0.625, 140 kvp, automated modulated mA 250–650, breath hold, contrast medium infusion of 4 m/s *via* an antecubital vein 18 g access).

According to reporting standards, the evaluation of anatomic and morphologic characteristics was performed in order to plan the EVAR procedure (8).

**Figure 1:**
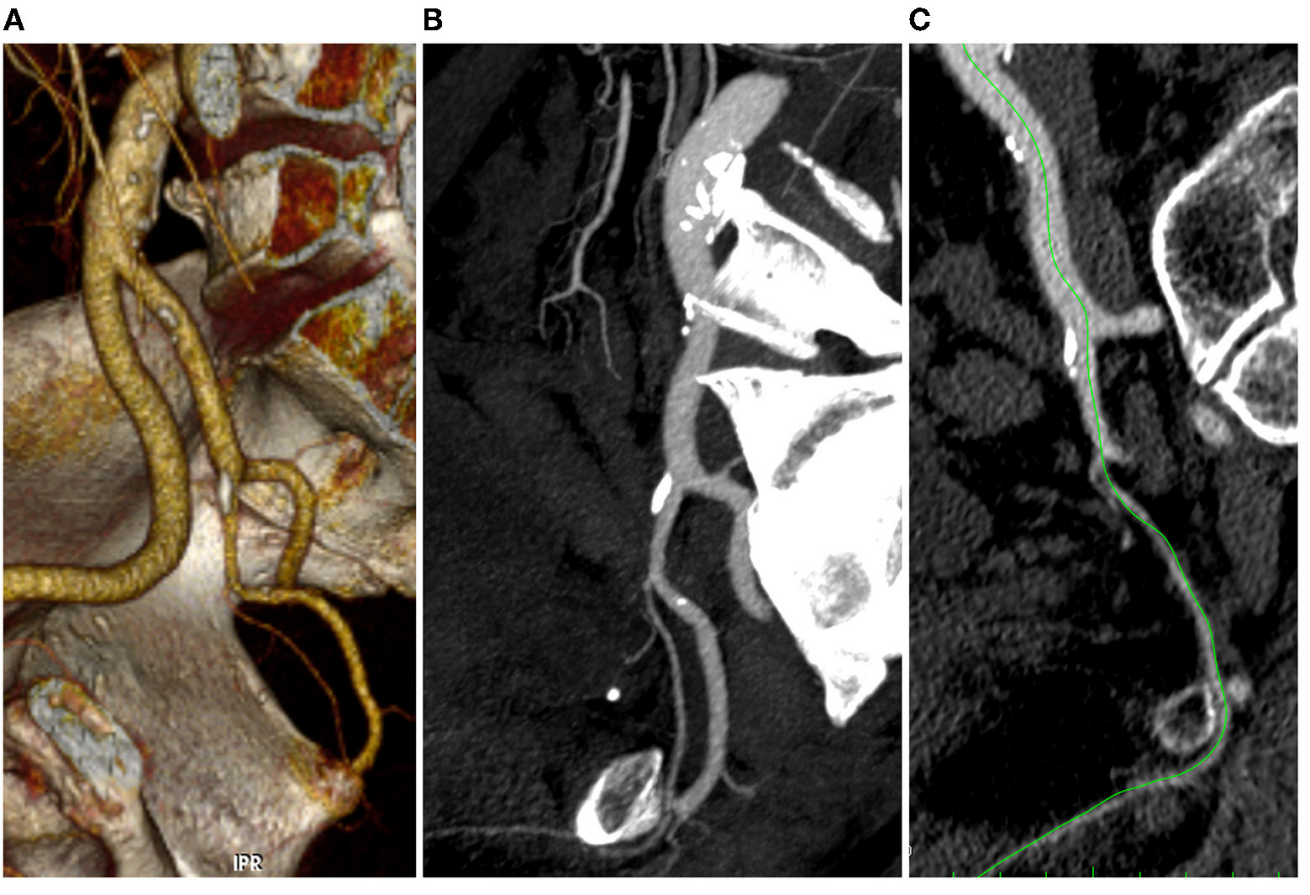
3D volume rendering **(A)**, multi-planar **(B)**, and center lumen line **(C)** reconstruction of the iliac-pelvic district (Anatomical Score 0-0-1-0).

Two operators independently reviewed the pelvic arterial tree of all patients without having information about IIEF-5 responses. A post-processing images analysis such as 3-D volume rendering, center-lumen line, and multi-planar reconstruction was performed in order to have a precise view of the hypogastric artery divisions (Figures 1A–C). Stenosis was evaluated using both CT images to have a morphological preliminary view and CT-Angio images using dedicated post-processing software in order to have the most correct arterial view and flow impairment. Stenosis percentages were calculated dividing residual lumen *vs*. native artery diameter (outer/outer layer).

An innovative score of pelvic arterial disease associated to AAA was defined. For this purpose, the iliac district was analyzed and divided it in four zones: (1) the inflow zone that corresponds to common iliac artery; (2) hypogastric zone I that includes the trunk of the hypogastric artery till its bifurcation in anterior and posterior division; (3) hypogastric zone II that includes the anterior division till the origin of the internal pudendal artery; (4) hypogastric zone III, the more distal, that includes the whole internal pudenda artery. The steno-occlusive disease was staged in four different groups: complete patency, <50, 50–79, and >80% stenosis. A score, ranging from 0 to 3 was associated to each lesion, where 0 was complete patency and 3 was >80% stenosis. The disease was considered diffuse if two or more zones had a score ≥1 per side.

**Figure 2:**
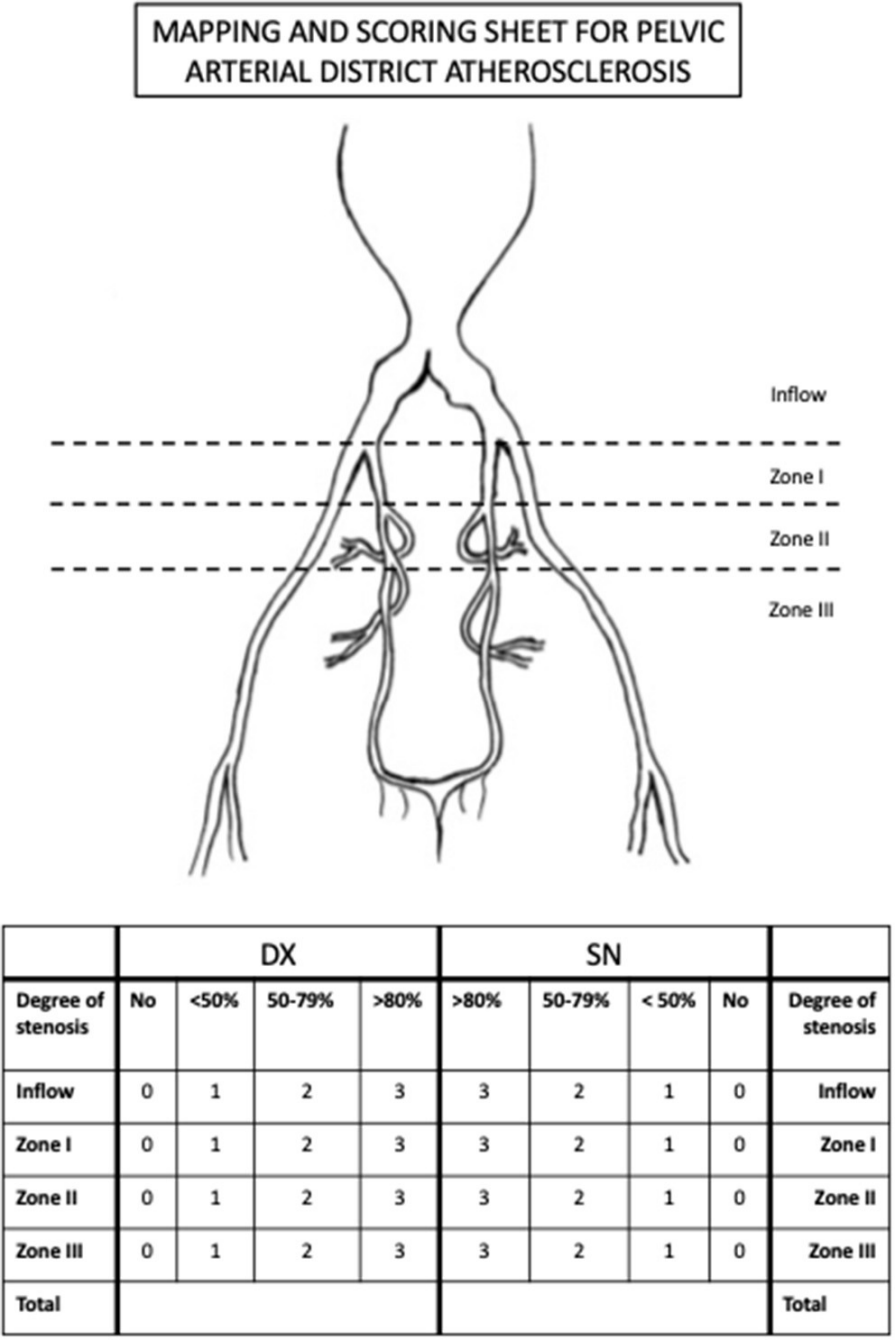
The “MAPPING AND SCORING SHEET” tool used for the evaluation of the steno-occlusive disease of the pelvic arterial tree.

The evaluation was repeated for both right and left arterial district. The outlined score ranges from 0 to 24 points. A dedicated “MAPPING AND SCORING SHEET” was designed in order to simplify the analysis (Figure 2).

According to this score, pelvic arterial tree disease is classified into 3 categories: severe (>12), moderate (4–11), and no significant disease (<3).

Arterial pelvic tree anatomy was also studied in order to detect anatomical variations according to Yamaki classification for both right and left hypogastric arteries (Groups A–D) (9).

Inter- and intra-observer variability was determined in all 25 CT scans.

### Statistical Analysis

Continuous data were shown as mean values ± SD. Categorical variables were expressed as fractions. Analysis of variance was used for independent tests to compare groups on continuous variables after demonstrating a normal distribution of the data.

IIEF-5 answers were graphically highlighted using a radar graph reporting the percentage of patients' answers (for the first question: very low, low, moderate, high, and very high; for questions no 2-3-5: almost/never/never, a few times, sometimes, most times, almost always/always; and for question no 4: extremely difficult, very difficult, difficult, slightly difficult, and not difficult).

Inter- and intra-observer variability was assessed using the Cohen's kappa test of concordance. A *k* value of 0.61–0.80 and 0.81–1.0 indicated good agreement and excellent agreement, respectively.

Correlation coefficients providing information about the strength and direction of a relationship between two continuous variables was calculated by the Pearson linear regression analysis. All statistical analyses were performed with GraphPad Prism 9.0 (GraphPad Software Inc., San Diego, Calif) and StatPlus Build 7.1.1 (AnalysisSoft Inc., Walnut, Calif).

## Results

### Study Population

A total of 31 patients underwent EVAR during the period study. Six of them were excluded to lack of Angio CT (3/6) and the rest due to the lack of consent. Median age was 74 ± 5.3 years. Baseline population characteristics were listed in Table 2. Particular attentions were dedicated to pharmacological anamnesis. In 3 cases both beta- and alpha blockers were used, in only two cases antidepressant therapy was chronically used. None of these patients have the history of prostatic interventions. AAA maximum diameter was 56 ± 6.3 mm. All patients underwent EVAR in elective setting using dedicated endografts.

**Table 2:** Baseline population characteristics.

**Clinical variables**	**Total population (*N* = 25)**
Age (mean ± SD)	74 ± 5.9
Diabetes mellitus (*N*; %)	3 (12%)
Coronary artery disease (*N*; %)	6 (24%)
Peripheral artery disease (*N*; %)	2 (8%)
Dyslipidemia (*N*; %)	15 (60%)
Hypertension (*N*; %)	17 (68%)
COPD (*N*; %)	5 (20%)
Chronic kidney disease (*N*; %)	13 (52%)
Atrial fibrillation (*N*; %)	1 (4%)
Smoke (*N*; %)	5 (20%)
Active smoker	2 (8%)
Former smoker	
BMI > 25 (*N*; %)	1 (4%)
Alcohol (>30 glasses/week) (*N*; %)	0 (0%)
Drugs assumption (*N*; %)	
Alfa-blockers	7 (28%)
Beta-blockers	7 (28%)
Statin	15 (60%)
Antidepressant	2 (8%)
Benzodiazepine	1 (4%)
Carbamazepine	1 (4%)
Finasteride	1 (4%)
Thiazide diuretics	3 (12%)

*SD, Standard Deviation; COPD, Chronic Obstructive Pulmonary Disease; BMI, Body Mass Index*.

### Erectile Dysfunction Outcomes

Only 2 patients (8%) declared to have discussed sexual issues with a physician in the past.

**Figure 3:**
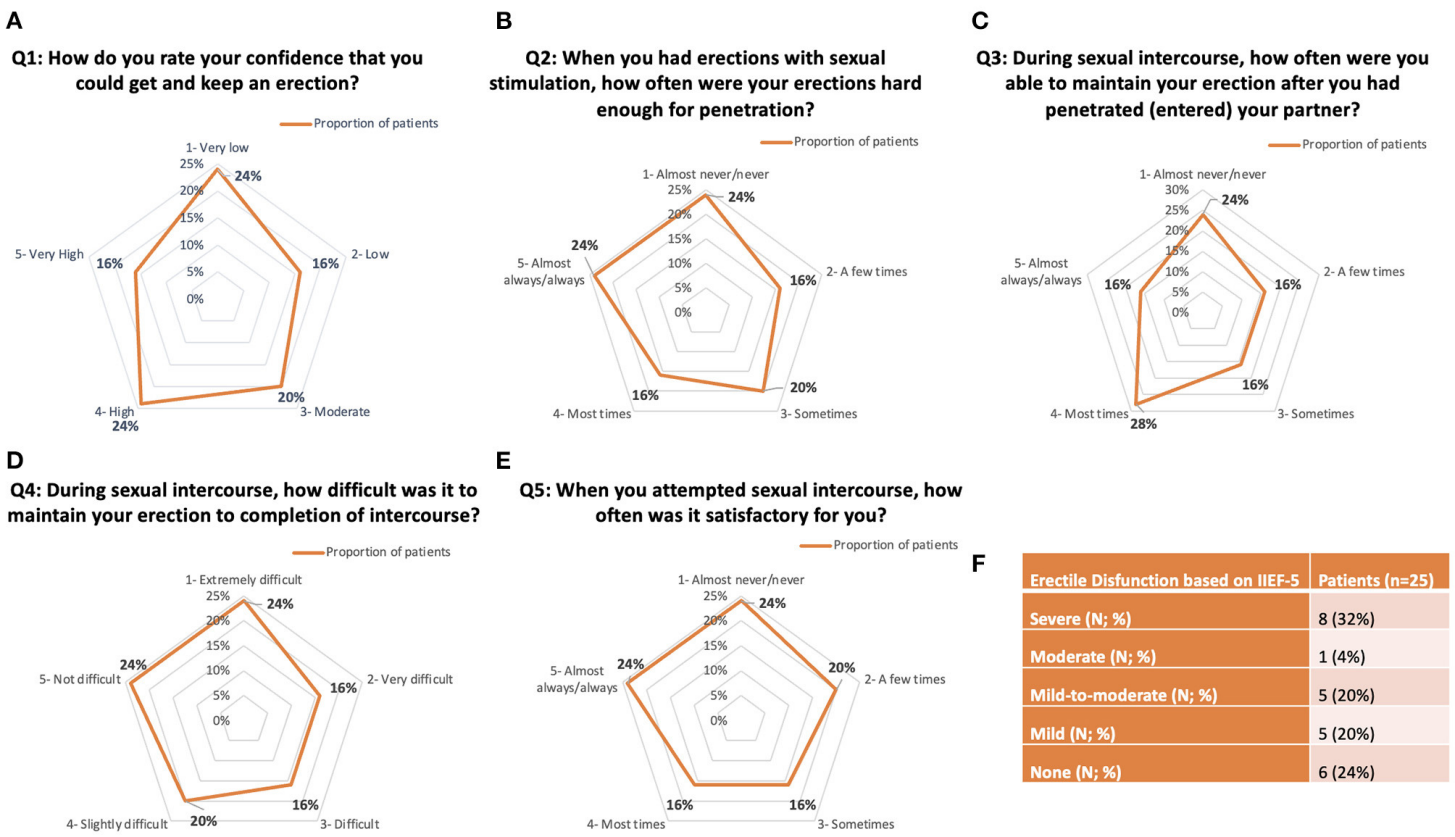
Detailed answers to IIEF-5 questionnaire. **(A–E)** Radar graph representations of the five IIEF-5 questions. **(F)** Summary of Erectile Disfunction outcomes based on the IIEF-5 answers.

All patients completed the IIEF-5 questionnaire. IIEF-5 average score was 14.8 ± 7.1. Eight cases (32%) had severe ED (IIEF-5 score 5–7); one case (4%) had moderate ED (IIEF-5 score 8–11); 4 patients (16%) had mild-to-moderate ED (IIEF-5 score 12–16); 6 patients (24%) had mild ED (IIEF-5 score 17–21), and only 6 (24%) patients had no ED (score > 22). Detailed outcomes were highlighted in Figure 3.

### Pelvic Arterial Tree Evaluation

Before starting the population analysis, two operators (GdD and EP) trained with 10 CT angiography evaluations of previous patients not included in this study, in order to identify the pelvic tree vascularization patterns.

Then, all CT angiography exams were independently reviewed by the two operators without knowing the IIEF-5 score of each patient enrolled.

The inter-observer variability was very good for the identification of the pelvic steno-occlusive disease at any of the 4 zones: overall evaluation *k* = 0.9; zone I *k* = 0.99; zone II *k* = 092; zone III *k* = 0.85; and zone 4 *k* = 0.91.

The intra-observer agreement, evaluated by asking each observer to assess the images twice with a 7-day interval, was excellent (*k* = 0.95).

Meantime to fill a single patient sheet was 9 ± 3 min.

Table 3 summarizes the “MAPPING AND SCORING SHEET” detailed results.

**Table 3:** Anatomical localization of steno-occlusive disease of pelvic arterial tree following the “MAPPING AND SCORING SHEET” grading.

	**Right district**				**Left district**				
**Degree of stenosis**	**No**	**<50%**	**50-79%**	**>80%**	**>80%**	**50-79%**	**<50%**	**No**	**Degree of stenosis**
Inflow	0/25 (0%)	3/25 (12%)	0/25 (0%)	0/25 (0%)	0/25 (0%)	1/25 (4%)	4/25 (16%)	0/25 0/%)	Inflow
Zone I	0/25 (0%)	14/25 (56%)	5/25 20%	2/25 (8%)	2/25 (8%)	4/25 (16%)	13/25 (52%)	0/25 (0%)	Zone I
Zone II	0/25 (0%)	8/25 (32%)	10/25 (40%)	3/25 (12%)	4/25 (16%)	9/25 (36%)	9/25 (36%)	0/25 (0%)	Zone II
Zone III	0/25 (0%)	5/25 (20%	8/25 (32%)	2/25 (8%)	5/25 (20%)	5/25 (20%)	5/25 (20%)	0/25 (0%)	Zone III

According to our innovative score of pelvic arterial disease, the average score was 7.9 ± 4.5.

Pelvic disease was considered severe-moderate in 20 cases (80%) and not significant in 20% (5 cases).

**Figure 4:**
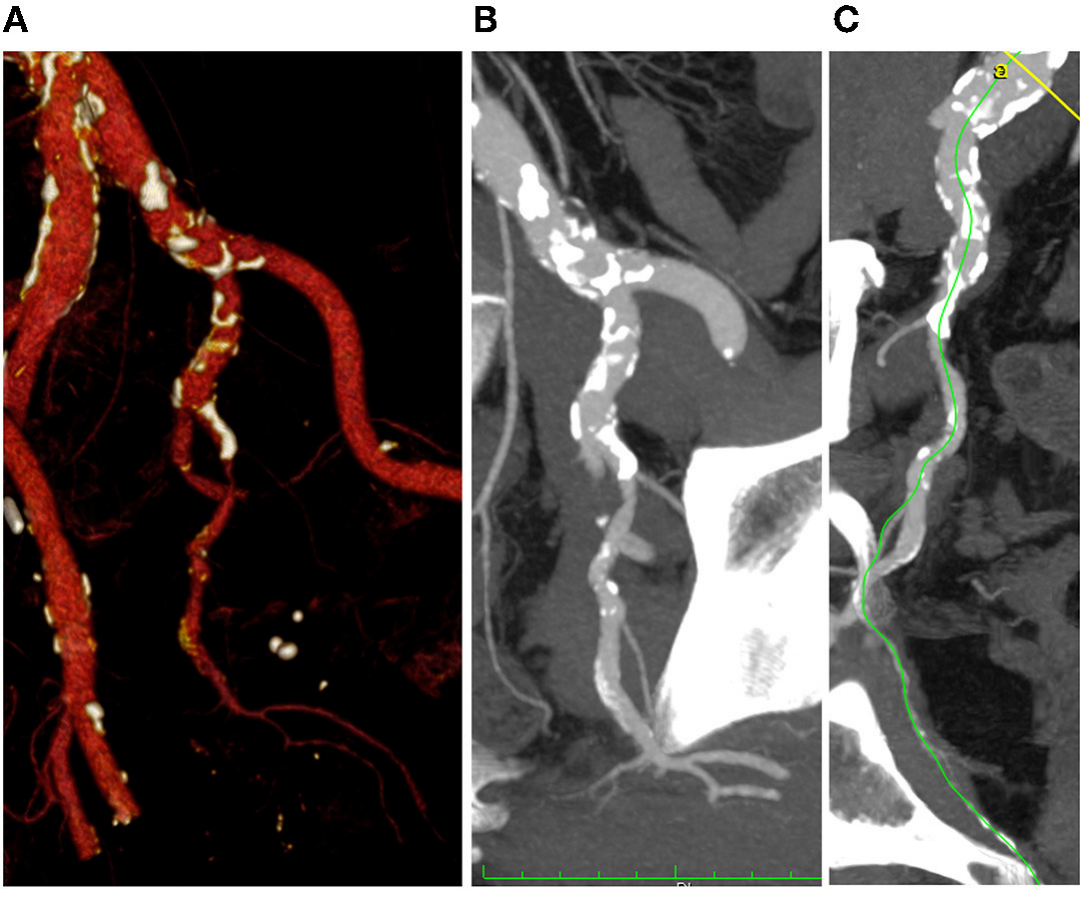
3D volume rendering **(A)**, multi-planar **(B)**, and center lumen line **(C)** reconstruction of the iliac-pelvic district (Anatomical Score 1-1-3-2).

The most pathological segment appeared to be zone II (mean score 1.52), the inflow zone corresponding to common iliac artery was the lesser pathologic (mean score 0.18). Figure 4 shows an example of significant pelvic arterial disease.

The steno-occlusive disease was localized in 16% (8/50) and diffuse in 80% (40/50). In one patient both left and right iliac district was not characterized by atherosclerotic disease.

Anatomical study of the internal iliac artery district according to the Yamaki classification revealed that most hypogastric branching types were Group A (34/50, 68%), followed by Group B (9/50, 18%), and Group C (7/50, 14%).

### Erectile Dysfunction and Pathological Arterial Tree Correlation

The IIEF-5 score and the anatomical score were analyzed to estimate any correlation between the severity of the anatomic disease and the severity of ED.

**Figure 5:**
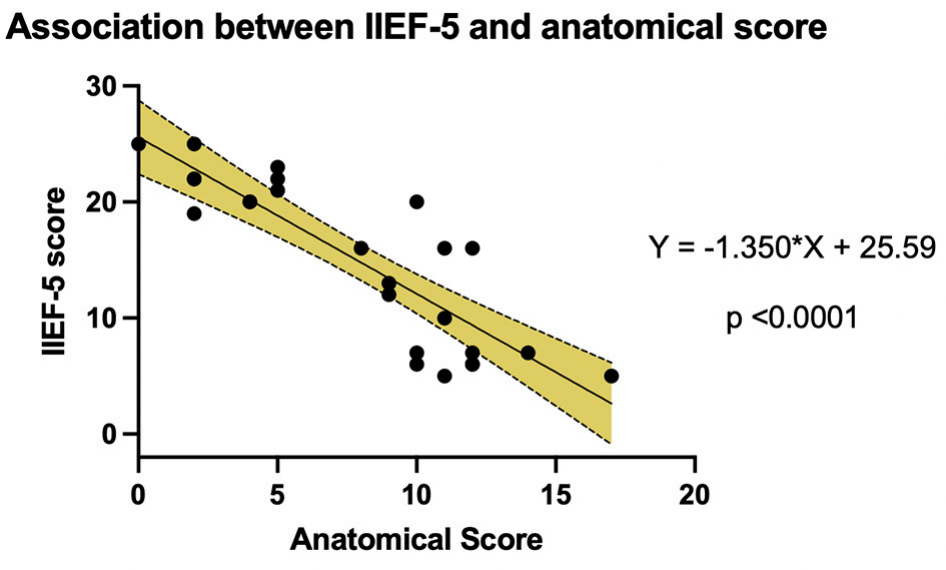
Linear regression analysis correlating IIEF-5 outcomes and the anatomical score.

The data were examined using linear regression analysis (Figure 5). The analysis confirmed the hypothesis that a more diseased pelvic arterial tree was correlated to a more severe ED. The linear regression line was defined by the equation “*Y* = −1.350^*^ × + 25.59” (slope *CI*: −1.7 to −1.0, *p* < 0.0001).

## Discussion

The main considerations from the analysis of the vasculogenic ED in an AAA population by the proposed investigation are:

1. Three out of 4 AAA patients had ED at IIEF-5 survey.
2. Most patients had never discussed sexual issue with a physician.
3. Four out of 5 AAA patients had an associated pelvic arterial disease.
4. Arterial lesions were often diffuse and included mainly hypogastric zone II.

Erectile dysfunction is defined as the recurrent or persistent inability to achieve or maintain an erection for satisfactory intercourse to occur. The deterioration of erectile function is age-related and it was first described by Kinsey et al. (10). Recently ED has been recognized as an organic and physiologic defect affecting the penile circulation as part of a more global vascular disease (11). In recent years, the presence of ED has been also considered as the first manifestation of multidistrict arteriopathy as CAD and PAD, but rarely has been evaluated as associated to AAA. Hypercholesterolemia, hypertension, cigarette smoking, and other common atherosclerotic risk factors all promote occlusive disease in various arterial beds and ultimately lead to a form of vasculogenic ED. Diabetes Mellitus has also been considered a significant risk factor for ED. On the other hand, its role in the aortic aneurysm formation and progression is still debated; in fact, several studies have highlighted that DM has a protective influence on AAA evolution. In this light, ED prevalence in AAA patients could be lower with respect to CAD/PAD patients for whom DM represents a major risk factor (12, 13). Impaired arterial inflow to the penis due to occlusion or narrowing of the common iliac arteries, internal iliac arteries, and the internal pudendal arteries and their distal branches may cause decreased penile rigidity during erection as well as prolongation of the time to peak erection.

Several studies have investigated the interplay of ED and CAD. These two conditions are distinct manifestations of the same degenerative arterial disease, highlighted by several shared independent risk factors. There is a growing body of literature supporting ED as the likely index diagnosis, preceding the development of symptomatic CAD. Various papers have evidenced that the ED could anticipate the CAD presentation for 2–5 years (14–16).

Reasonably, ED has been linked to PAD and Carotid artery disease, respectively. Polonsky et al. reported that patients with ED were found to have significantly more PAD than men without ED (32 *vs*. 16%), and there was a stepwise increase in the prevalence of PAD with increasing ED severity (28% of men with mild ED, 33% with moderate ED, and 40% with severe ED). These results were confirmed also by the multivariate logistic regression analysis in which ED was an independent predictor of PAD (17). Carotid artery intima-media thickness was also related to ED, in fact, some papers highlighted that patients with ED have greater carotid artery intima-media thickness and higher rates of carotid plaque prevalence (18–21) especially in the presence of metabolic syndrome and chronic kidney disease.

We set up exploratory research on the prevalence of ED in the AAA population. By definition, an exploratory study investigates a problem which is not clearly defined and cannot provide conclusive results. However, our investigation clearly revealed that ED is present in a considerable number of patients with AAA undergoing EVAR, and that vasculogenic origin of sexual dysfunction may be the cause.

Open elective AAA repair (OR) has a clear and documented impact on sexual function in the postoperative period (22–25). Key points for reducing sexual dysfunction after OR are to preserve blood flow to the internal iliac arteries and avoid injury to the autonomic nerves around the aortoiliac bifurcation. Beyond these basic principles, however, the impact of vascular surgery on sexual function is ignored, mainly because the hypogastric artery distribution, and in particular the pudenda artery disease, is rarely assessed.

Endovascular aneurysm repair has been established as fundamental alternative to OR, allowing to treat patients with a minimally invasive technique. Time has permitted to develop new technologies capable of threating difficult anatomies (26–29) and very high-risk patients (30). A similar sexual dysfunction has been reported after EVAR, but the recovery to the preoperative level was faster with EVAR than after OR (31).

In all cases for patients undergoing AAA repair, little is known about the importance and frequency of any sexual dysfunction. Preoperatively given information is typically lacking details on sexual function, and correlation to pelvic vascularization is poorly evaluated.

The current guidelines from the American Urological Association (32) recommend that men presenting with symptoms of ED should undergo thorough medical, sexual, and psychosocial history; a physical examination; and selective laboratory testing. Then, clinicians should counsel men with ED who have comorbidities known to negatively affect erectile function that lifestyle modifications, such as changes in diet and increased physical activity, improve overall health, and may improve erectile function. Oral phosphodiesterase type 5 inhibitor is then typically prescribed, and instructions should be provided to maximize benefit/efficacy. In addition, in the case of contemporary disease, CAD or PAD is trying to maximize the adjuvant medical therapy (33) that could be useful for the multilevel atherosclerotic disease.

However, when oral ED therapy fails, subsequent therapies are progressively invasive and include intracavernosal injections, intraurethral suppositories, vacuum erection devices, and penile prosthesis implantation.

Arterial intervention is only recommended for younger patients with isolated vascular injuries, typically from previous traumatic experiences.

According to our results, the presence of a proximal fixed obstruction to arterial flow may be present in patients with AAA, who are typically older than those recommended for arterial intervention by the guidelines.

Our exploratory investigation suggests that a simple “MAPPING AND SCORING SHEET” may reveal vasculogenic origin of ED in a certain number of patients presenting with AAA.

After a brief learning curve to recognize the pelvic vasculature, the overview of arterial pelvic disease for each patient was obtained with an excellent inter- and intra-observer agreement (*k* > 0.81). This may suggest to applying the proposed sheet as a tool to investigate ED in AAA patients, without adding extra investigation, with a reasonable examination time.

Interventional treatment of atherosclerotic disease in the pelvic district, therefore, may offer another approach to ED refractory to current first-line therapies, but additional investigation is required prior to universal endorsement.

These data support that all AAA patients who typically are evaluated with a high-quality angio-CT have a good and unplanned opportunity to investigate pelvic vasculature and discuss with physician about sexual dysfunction.

### Limitations

The main limitation of our investigation was the small patient population, not allowing for adjustment for possible confounders such as age, diabetes mellitus, medication, active smoking, and psychiatric disorders. A clear limitation is when only patient self-reporting data are taken into consideration in the absence of objective measures. Although we recognize that our analyses are only partially based on patient self-reporting data, anatomical data from angio-CT provided objective measures of vascular anatomy and penile blood flow, potential surrogate variables for sexual performance. Still ED may be classified as psychological, organic (vasculogenic, neurogenic, or endocrinologic), or drug-induced, and our investigation was not designed to evaluate the risk factors in the AAA population. So, we recognize that some biases may have influenced our results and analyses.

## Conclusion

Although typically unreported, the prevalence of ED associated to AAA was found to be high. A vasculogenic origin of ED in patients with AAA is plausible and may be easily confirmed by the evaluation of arterial pelvic distribution at angio-CT performed for EVAR planning. Our proposed “MAPPING AND SCORING SHEET” may help to identify the vasculogenic origin of ED in AAA patients.

## Data Availability Statement

The raw data supporting the conclusions of this article will be made available by the authors, without undue reservation.

## Ethics Statement

Ethical review and approval was not required for the study on human participants in accordance with the local legislation and institutional requirements. The patients/participants provided their written informed consent to participate in this study.

## Author Contributions

GD, EP, and GP: conception and design. GD, EP, and GG: analysis and interpretation. BG, GC, and GF: data collection. GD and EP: writing the manuscript. All the authors critical revision and approval of the manuscript.

## Conflict of Interest

The authors declare that the research was conducted in the absence of any commercial or financial relationships that could be construed as a potential conflict of interest.

## Publisher's Note

All claims expressed in this article are solely those of the authors and do not necessarily represent those of their affiliated organizations, or those of the publisher, the editors and the reviewers. Any product that may be evaluated in this article, or claim that may be made by its manufacturer, is not guaranteed or endorsed by the publisher.

## Abbreviations

AAA: Abdominal Aortic Aneurysm
AF: Atrial Fibrillation
CAD: Coronary Artery Disease
CHF: Congestive Heart Failure
COPD: Chronic Obstructive Pulmonary Disease
CT: Computed Tomography
DM: Diabetes Mellitus
ED: Erectile Dysfunction
EVAR: Endovascular Aneurysm Repair
IIEF-5: International Index of Erectile Function-5
OR: Open Repair
PAD: Peripheral Artery Disease.

## References

[1] Siroky MB Azadzoi KM. Vasculogenic erectile dysfunction: newer therapeutic strategies. J Urol. (2003) 170:S24–9. 10.1097/01.ju.0000075361.35942.17 12853769

[2] Willke RJ Yen W Parkerson GR Jr Linet OI Erder MH Glick HA. Quality of life effects of alprostadil therapy for erectile dysfunction: results of a trial in Europe and South Africa. Int J Imp Res. (1998) 10:239–46. 10.1038/sj.ijir.3900364 9884920

[3] Feldman HA Goldstein I Hatzichristou DG Krane RJ McKinlay JB. Impotence and its medical and psychosocial correlates: re- sults of the Massachusetts Male Aging Study. J Urol. (1994) 151:54–61. 10.1016/S0022-5347(17)34871-1 8254833

[4] Meller SM Stilp E Walker CN Mena-Hurtado C. The link between vasculogenic erectile dysfunction, coronary artery disease, and peripheral artery disease: role of metabolic factors and endovascular therapy. J Invasive Cardiol. (2013) 25:313–9. 23735361

[5] Pettersson M Mattsson E Bergbom I. Prospective follow-up of sexual function after elective repair of abdominal aortic aneurysms using open and endovascular techniques. J Vasc Surg. (2009) 50:492–9. 10.1016/j.jvs.2009.02.245 19700089

[6] Rosen RC Riley A Wagner G Osterloh IH Kirkpatrick J Mishra A. The international index of erectile function (IIEF): a multidimensional scale for assessment of erectile dysfunction. Urology. (1997) 49:822–30. 10.1016/S0090-4295(97)00238-0 9187685

[7] Rhoden EL Telöken C Sogari PR Vargas Souto CA. The use of the simplified International Index of Erectile Function (IIEF-5) as a diagnostic tool to study the prevalence of erectile dysfunction. Int J Impot Res. (2002) 14:245–50. 10.1038/sj.ijir.3900859 12152112

[8] Chaikof EL Fillinger MF Matsumura JS Rutherford RB White GH Blankensteijn JD. Identifying and grading factors that modify the outcome of endovascular aortic aneurysm repair. J Vasc Surg. (2002) 35:1061–6. 10.1067/mva.2002.123991 12021728

[9] Yamaki K Saga T Doi Y Aida K Yoshizuka M. A statistical study of the branching of the human internal iliac artery. Kurume Med J. (1998) 45:333–40. 10.2739/kurumemedj.45.333 9914720

[10] Kinsey AC Pomeroy WB Martin CE. Sexual Behavior in the Human Male. Philadelphia, PA: W.B. Saunders (1948). p. 610–66.

[11] Johannes CB Araujo AB Feldman HA Derby CA Kleinman KP McKinlay JB. Incidence of erectile dysfunction in men 40 to 69 years old: longitudinal results from the Massachusetts male aging study. J Urology. (2000) 163:460–3. 10.1016/S0022-5347(05)67900-1 10647654

[12] Raffort J Lareyre F Clément M Hassen-Khodja R Chinetti G Mallat Z. Diabetes and aortic aneurysm: current state of the art. Cardiovasc Res. (2018) 114:1702–13. 10.1093/cvr/cvy174 30052821 PMC6198737

[13] Kouidrat Y Pizzol D Cosco T Thompson T Carnaghi M Bertoldo A. High prevalence of erectile dysfunction in diabetes: a systematic review and meta-analysis of 145 studies. Diabet Med. (2017) 34:1185–92. 10.1111/dme.13403 28722225

[14] Virag R Bouilly P Frydman D. Is impotence an arterial disorder? A study of arterial risk factors in 440 impotent men. Lancet. (1985) 1:181–4. 10.1016/S0140-6736(85)92023-9 2857264

[15] Montorsi P Ravagnani PM Galli S Rotatori F Veglia F Briganti A. Association between erectile dysfunction and coronary artery disease. Role of coronary clinical presentation and extent of coronary vessels involvement: the cobra trial. Eur Heart J. (2006) 27:2632–9. 10.1093/eurheartj/ehl142 16854949

[16] Montorsi P Ravagnani PM Galli S Briganti A Salonia A Dehò F. Association between erectile dysfunction and coronary artery disease: a case report study. J Sex Med. (2005) 2:575–82. 10.1111/j.1743-6109.2005.00084.x 16422857

[17] Polonsky TS Taillon LA Sheth H Min JK Archer SL Ward RP. The association between erectile dysfunction and peripheral arterial disease as determined by screening ankle-brachial index testing. Atherosclerosis. (2009) 207:440–4. 10.1016/j.atherosclerosis.2009.05.005 19501825

[18] Unal M Aksoy DY Aydin Y Tanriover MD Berker D Karakaya J Guler S. Carotid artery intima-media thickness and erectile dysfunction in patients with metabolic syndrome. Med Sci Monit. (2014) 20:884–8. 10.12659/MSM.889771 24869934 PMC4049947

[19] Stolic RV Bukumiric ZM. Intima-media thickness of carotid arteries and erectile dysfunction in hemodialysis patients. Hemodial Int. (2010) 14:510–4. 10.1111/j.1542-4758.2010.00493.x 20955285

[20] Yao FJ Zhang YD Wan Z Li W Lin H Deng CH. Erectile dysfunction is associated with subclinical carotid vascular disease in young men lacking widely-known risk factors. Asian J Androl. (2018) 20:400–4. 10.4103/aja.aja_73_17 29442076 PMC6038168

[21] Feldman DI Cainzos-Achirica M Billups KL DeFilippis AP Chitaley K Greenland P. Subclinical Vascular Disease and Subsequent Erectile Dysfunction: The Multiethnic Study of Atherosclerosis (MESA). Clin Cardiol. (2016) 39:291–8. 10.1002/clc.22530 27145089 PMC4879072

[22] Xenos ES Stevens SL Freeman MB Pacanowski JP Cassada DC Goldman MH. Erectile function after open or endovascular abdominal aortic aneurysm repair. Ann Vasc Surg. (2003) 17:530–8. 10.1007/s10016-003-0058-2 14508665

[23] Regnier P Lareyre F Hassen-Khodja R Durand M Touma J Raffort J. Sexual dysfunction after abdominal aortic aneurysm surgical repair: current knowledge and future directions. Eur J Vasc Endovasc Surg. (2018) 55:267–80. 10.1016/j.ejvs.2017.11.028 29292207

[24] Lee ES Kor DJ Kuskowski MA Santilli SM. Incidence of erectile dysfunction after open abdominal aortic aneurysm repair. Ann Vasc Surg. (2000) 14:13–9. 10.1007/s100169910003 10629258

[25] Lederle FA Freischlag JA Kyriakides TC Padberg FT Jr Matsumura JS Kohler TR. Open Versus Endovascular Repair (OVER) Veterans Affairs Cooperative Study Group. Outcomes following endovascular vs open repair of abdominal aortic aneurysm: a randomized trial. JAMA. (2009) 302:1535–42. 10.1001/jama.2009.1426 19826022

[26] de Donato G Pasqui E Mele M Panzano C Giannace G Setacci F. The use of a low-profile stent graft with a polymer ring sealing technology combined with bare renal stent (vent technique) in patients with juxtarenal aneurysm not eligible for open surgery and fenestrated endograft. J Vasc Surg. (2020) 71:1843–50. 10.1016/j.jvs.2019.06.220 31676183

[27] de Donato G Pasqui E Nano G Lenti M Mangialardi N Speziale F. LoLopro Registry Collaborators. Long-term results of low-profile stent grafts for treatment of infrarenal aortic aneurysms: Results from a retrospective multicenter registry. J Vasc Surg. (2021). 10.1016/j.jvs.2021.09.036. [Epub ahead of print]. 34634415

[28] de Donato G Pasqui E Panzano C Brancaccio B Grottola G Galzerano G Benevento D Palasciano G. The Polymer-Based Technology in the Endovascular Treatment of Abdominal Aortic Aneurysms. Polymers (Basel). (2021) 13:1196. 10.3390/polym13081196 33917214 PMC8068055

[29] de Donato G Pasqui E Panzano C Galzerano G Cappelli A Palasciano G. Early Experience with the New Ovation Alto Stent Graft in Endovascular Abdominal Aortic Aneurysm Repair. EJVES Vasc Forum. (2021) 54:7–12. 10.1016/j.ejvsvf.2021.11.003 34950916 PMC8671859

[30] Pasqui E de Donato G Giannace G Panzano C Setacci C Palasciano G. Management of abdominal aortic aneurysm in nonagenarians: a single-centre experience. Vascular. (2021) 29:27–34. 10.1177/1708538120936831 32611281

[31] Prinssen M Buskens E Nolthenius RP van Sterkenburg SM Teijink JA Blankensteijn JD. Sexual dysfunction after conventional and endovascular AAA repair: results of the DREAM trial. J Endovasc Ther. (2004) 11:613–20. 10.1583/04-1280R.1 15615551

[32] Burnett AL Nehra A Breau RH Culkin DJ Faraday MM Hakim LS. Erectile Dysfunction: AUA Guideline. J Urol. (2018) 200:633–41. 10.1016/j.juro.2018.05.004 29746858

[33] 33 de Donato G Benedetto F Stilo F Chiesa R Palombo D Pasqui E. Evaluation of Clinical Outcomes After Revascularization in Patients With Chronic Limb-Threatening Ischemia: Results From a Prospective National Cohort Study (RIVALUTANDO). Angiology. (2021) 72:480–9. 10.1177/0003319720980619 33406850

